# Seeing the air in detail: Hyperlocal air quality dataset collected from spatially distributed AirQo network

**DOI:** 10.1016/j.dib.2022.108512

**Published:** 2022-08-03

**Authors:** Richard Sserunjogi, Joel Ssematimba, Deo Okure, Daniel Ogenrwot, Priscilla Adong, Lillian Muyama, Noah Nsimbe, Martin Bbaale, Engineer Bainomugisha

**Affiliations:** AirQo, Department of Computer Science, Makerere University, Kampala, Uganda

**Keywords:** Air quality dataset, Sub-Saharan Africa, Air pollution, PM_2_*_._*_5_, PM_10_, Particulate matter

## Abstract

Air pollution is a major global challenge associated with an increasing number of morbidity and mortality from lung cancer, cardiovascular and respiratory diseases, among others. However, there is scarcity of ground monitoring air quality data from Sub-Saharan Africa that can be used to quantify the level of pollution. This has resulted in limited targeted air pollution research and interventions *e.g.* health impacts, key drivers and sources, economic impacts, among others; ultimately hindering the establishment of effective management strategies. This paper presents a dataset of air quality observations collected from 68 spatially distributed monitoring stations across Uganda. The dataset includes hourly PM_2_*_._*_5_ and PM_10_ data collected from low-cost air quality monitoring devices and one reference grade monitoring device over a period ranging from 2019 to 2020. This dataset contributes towards filling some of the data gaps witnessed over the years in ground level monitored ambient air quality in Sub-Saharan Africa and it can be useful to various policy makers and researchers.

## Specifications Table

**Table:** 

Subject	Environmental Science
Specific subject area	This paper focuses on providing ambient air quality (Particulate Matter (PM_2_*_._*_5_ and PM_10_)) dataset
Type of data	Table
How the data were acquired	The data was acquired from a network of air quality monitors deployed across Uganda. The dataset includes measurements obtained from AirQo [1] low-cost air quality monitors and a Met One Beta Attenuation Monitor Model 1022 reference grade monitor.
Data format	Processed
Description of data collection	Data was collected using AirQo low-cost monitors and a Met One Beta Attenuation Monitor from 68 spatially distributed monitoring stations across Uganda (see Fig. 1) over a period of time ranging from January 12th, 2019 to December 31st, 2020. Data from low-cost monitors was transmitted to a cloud platform every 90 seconds over a local cellular network. The raw data was re-sampled to an hourly frequency and the PM_2_*_._*_5_ and PM_10_ values were computed by averaging the observations from the dual sensors. Records having timestamps with missing or invalid measurements such as negative values and values greater than 500 were eliminated from the dataset.
Data source location	Country: Uganda
Data accessibility	Repository name: Mendeley Data Data identification number: doi:10.17632/r3hgnrb73w.2 Direct URL to data: http://dx.doi.org/10.17632/r3hgnrb73w.2 The dataset citation is in Ref [7] The AirQo network is evolving, new monitoring sites continue to be added to the network and new datasets become available. The new datasets are made available at AirQo platform(https://platform.airqo.net) for which access can be requested through the Request Access feature and consenting to the data usage agreement

**Fig. 1:**
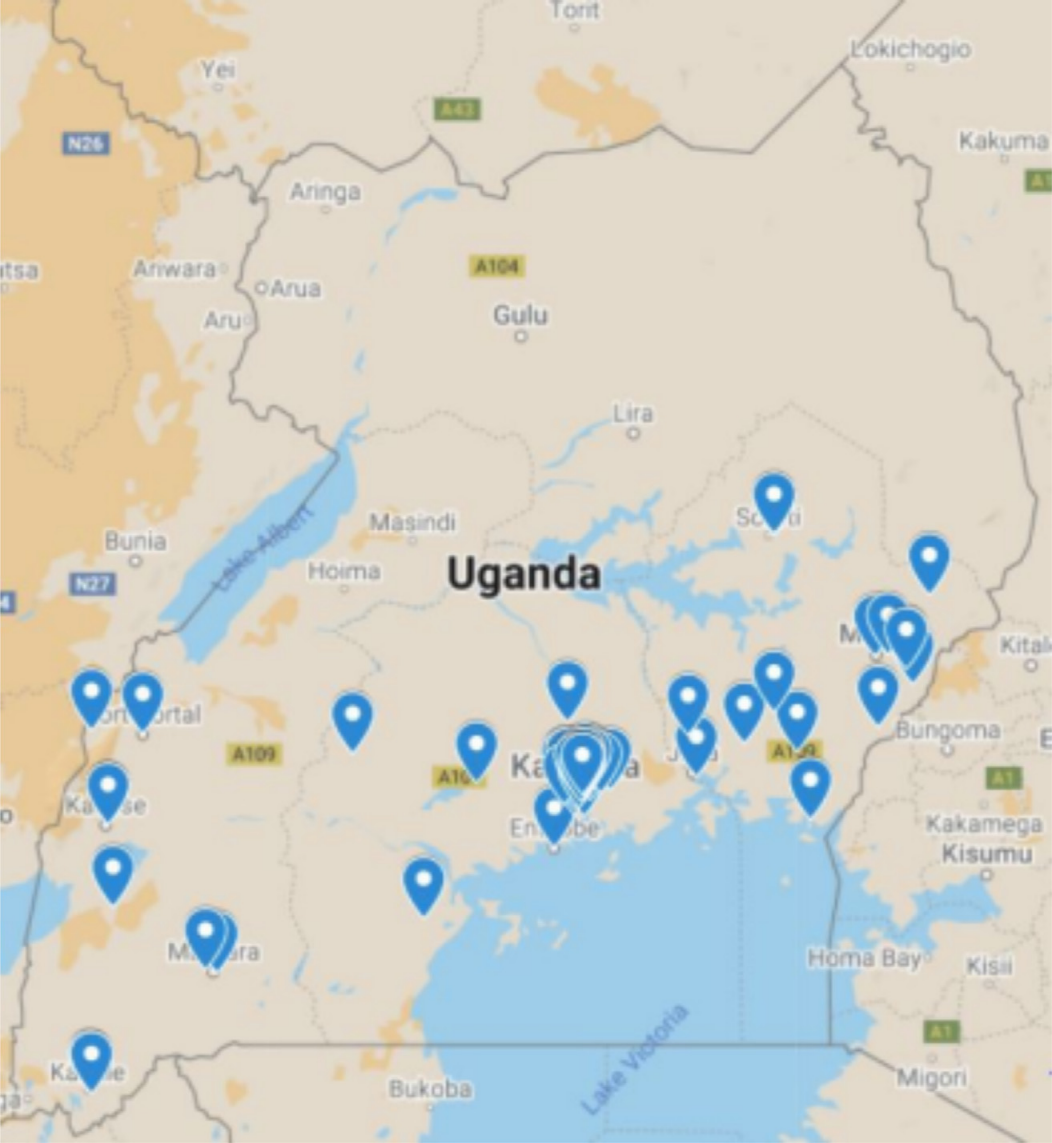
Map of Uganda showing the air quality monitoring sites

## Value of the Data

• The dataset is essential in filling some of the data gaps witnessed over the years in ground level monitored ambient air quality in Sub-Saharan Africa. In turn, policy makers can be guided in developing evidence-based air quality control strategies and prioritisation of air quality issues [2].
• Researchers and the academic community can utilise this dataset to carry out various research studies related to social economic impact of air pollution, and studies aiming at understanding the air pollution exposure risks [3].
• Researchers can use this dataset to facilitate the development of new & novel modelling algorithms in the air quality space *e.g.* forecasting, spatial temporal modelling and others.
• This dataset can be used as a baseline (ground truth) to highlight the potential of utilizing low-cost monitors in other countries/regions where air quality data is non-existent and probably model the air quality in those areas with similar characteristics as the region where the data was collected from.
• This dataset can be used in tracking the progress and implementation of World Health Organisation air quality guidelines [4].
• This dataset can be fused with other datasets such as satellite data for environmental and air quality modelling.

## 1 Data Description

**Fig. 2:**
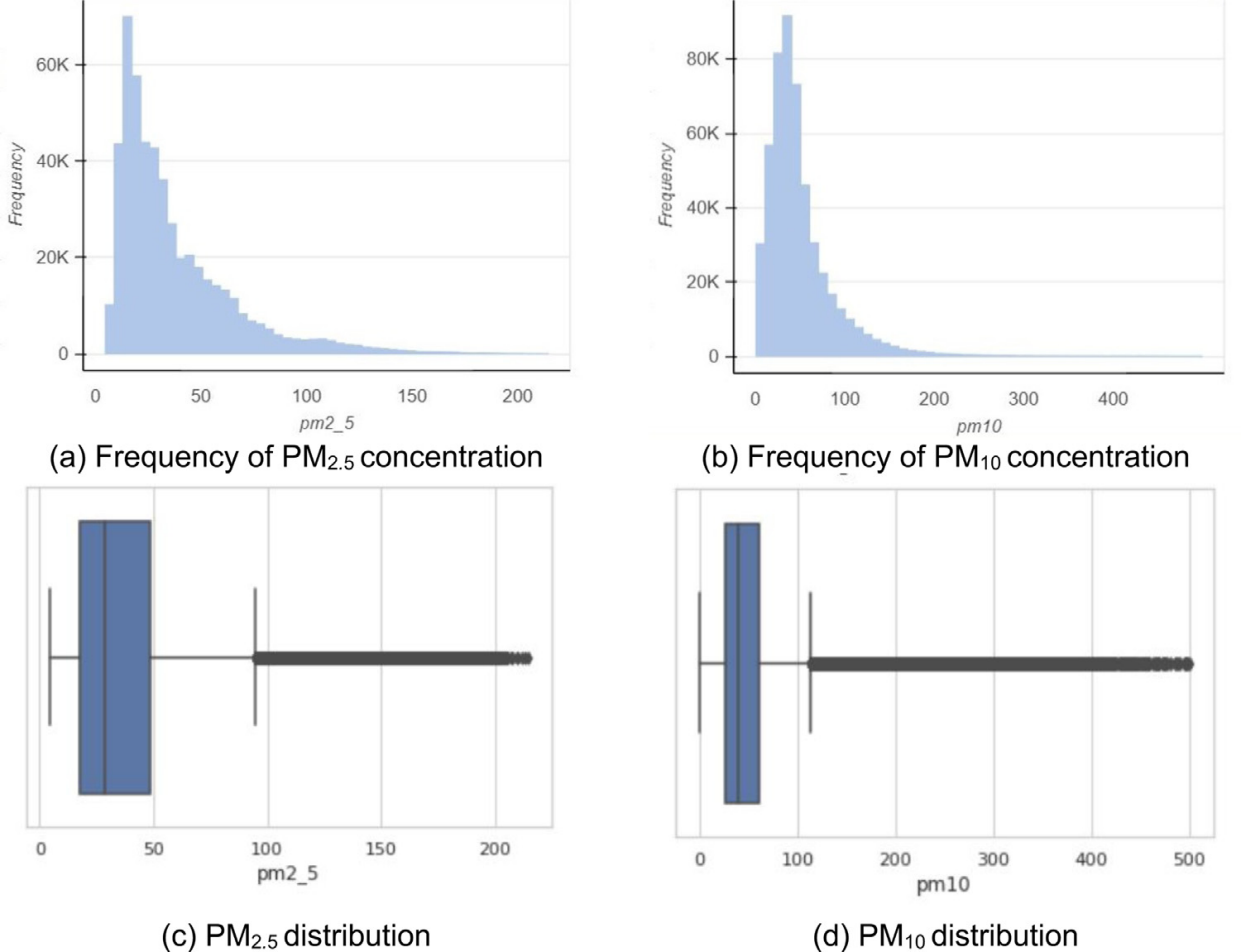
Distribution of PM_2_*_._*_5_ and PM_10_ over the network

The air quality dataset presented in this article comprises of records containing timestamp in UTC, PM_2_*_._*_5_ concentrations in µg*/m*^3^, PM_10_ in µg*/m*^3^, site id which uniquely identifies a monitoring site and the site coordinates (latitude, longitude). It contains 506164 records from low-cost monitors and 3,364 records from the reference grade monitor. The data from the various monitoring devices have varying start dates since they were deployed on different dates as the network is continuously being expanded. The mean PM_2_*_._*_5_ and PM_10_ concentrations from the low-cost monitors dataset are 37.39 µg*/m*^3^ and 49.61 µg*/m*^3^ respectively. Table 1 and Fig. 2 show the statistical summary of the data from the low-cost monitors. Table 2 shows the statistical summary of the data from the reference grade monitor.

**Table 1:** Statistical summary of the data from the low-cost monitoring devices

	Mean	STD	Min	25%	50%	75%	Max
PM_2_*_._*_5_	37.39	27.99	4.77	17.54	28.50	48.50	214.43
PM_10_	49.61	38.51	1.00	26.07	40.05	60.80	499.45

**Table 2:** Statistical summary of the data from the reference grade monitor

	Mean	STD	Min	25%	50%	75%	Max
PM_2_*_._*_5_	36.99	22.86	1.00	20.90	31.30	48.40	183.40

## 2 Experimental Design, Materials and Methods

The data presented in the article was collected from a network of AirQo [1] low-cost monitors and one reference grade monitor. The monitoring sites were selected with the aim of monitoring pollution variations for diverse physical environments in the selected urban centres *i.e.* population distribution (high population density vs low population density), commercial centres vs residential areas, urban background vs non-urban background, proximity to emission sources *e.g.* road network, industries and others. The monitoring site with the reference grade monitor is an institutional setting with a resident population of over 5000, having paved roads and vegetation canopies. It's located about 0.6 km from a major road and is 1237.39 meters above sea level. The reference grade monitor is a Met One Beta Attenuation Monitor Model 1022 [5, 6] which uses the principle of beta ray attenuation to continuously monitor particulate matter. It is configured to measure and record hourly PM_2_*_._*_5_ concentration. On the other hand, the low-cost monitors use laser scattering technique and utilise dual Plantower Sensors (PMS 5003) [2]. These devices measure PM_2_*_._*_5_ and PM_10_ with an effective range of 0-500µg*/m*^3^ as well as the device location coordinates. Thereafter, the measured data is transmitted to a cloud platform every 90 seconds over a local cellular network. The raw data from low-cost monitors is then extracted from the cloud platform and re-sampled to an hourly frequency. The PM_2_*_._*_5_ and PM_10_ values are computed by averaging the observations from the dual sensors. Records having timestamps with missing or invalid measurements such as negative values and values greater than 500 are eliminated from the dataset. The raw measurements from the low-cost monitors are calibrated by applying appropriate machine learning models trained on data from collocated low-cost and reference-grade monitors [8]. These models were validated through cross-unit and cross-site validation. PM_2.5_ measurements were calibrated by applying random forest model which improved the RMSE & MAE from 18.58µg/m^3^ to 7.22µg/m^3^ and 14.60µg/m^3^ to 4.60µg/m^3^ respectively when compared to the collocated reference monitor readings. PM_10_ measurements were calibrated using the lasso regression model which improved RMSE and MAE from 13.40µg/m^3^ to 7.91µg/m^3^ and 11.32µg/m^3^ to 6.01µg/m^3^ respectively. The statistical summaries for the processed dataset were then computed.

## Ethics Statements

To preserve the privacy of individuals and institutions hosting the monitoring devices, random coordinate distance preserving transformations were done on the actual coordinates of the monitoring sites. The distance between the transformed coordinates and actual coordinates varies between 50 and 110 metres with an average of 78.35 metres

## CRediT Author Statement

**Richard Sserunjogi:** Conceptualization, Data curation, Writing – original draft preparation, Methodology; **Joel Ssematimba:** Methodology, Software, Data curation; **Daniel Ogenrwot:** Writing – reviewing & editing, Software; **Priscilla Adong:** Data curation, Writing – review & editing; **Lillian Muyama:** Data curation, Writing – review & editing; **Noah Nsimbe:** Software, Data curation; **Martin Bbaale:** Software; **Deo Okure:** Project administration, Writing – review and editing; **Engineer Bainomugisha:** Supervision, Conceptualization, Project administration, Writing – review and editing.

## Declaration of competing Interest

The authors declare that they have no known competing financial interests or personal relationships which have or could be perceived to have influenced the work reported in this article.

All the authors declare that their affiliation to AirQo and Makerere University has not influenced the work reported in this paper.

## Data Availability

Seeing the air in detail: hyperlocal air quality dataset collected from spatially distributed AirQo network (Original data) (Mendeley Data).

## References

[1] AirQo, Airqo, africa's air quality network. https://www.airqo.net. Accessed April 28, 2022.

[2] Okure D., Ssematimba J., Sserunjogi R., Gracia N.L., Soppelsa M.E., Bainomugisha E. (2022). Characterization of ambient air quality in selected urban areas in uganda using low-cost sensing and measurement technologies. Environ. Sci. Technol..

[3] Chen J., Hoek G. (2020). Long-term exposure to pm and all-cause and cause-specific mortality: a systematic review and meta-analysis. Environ. Int..

[4] Weltgesundheitsorganisation, W. H. Organization, and E. C. for Environment (2021).

[5] Met One Instruments Inc, Bam-1022 beta attenuation mass monitor - met one instruments. https://metone.com/products/bam-1022/. Accessed April 28, 2022.

[6] Gobeli D., Schloesser H., Pottberg T. (2008). The Air & Waste Management Association (A&WMA) Conference.

[7] Sserunjogi R., J Sematimba, Okure D., Ogenrwot D., Adong P., Muyama L., Nsimbe N., Bbaale M., Bainomugisha E. (2022). Seeing the air in detail: hyperlocal air quality dataset collected from spatially distributed AirQo network. Mendeley Data.

[8] Adong P., Bainomugisha E., Okure D., Sserunjogi R. (2022). Applying machine learning for large scale field calibration of low-cost PM2.5 and PM10 air pollution sensors. Applied AI Letters.

